# The Impact of Workplace Spirituality and Psychological Capital on Elementary School Teachers’ Motivation

**DOI:** 10.3390/bs14100881

**Published:** 2024-10-01

**Authors:** Shwu-Ming Wu

**Affiliations:** Department of Human Resource Development, National Kaohsiung University of Science and Technology, Kaohsiung 824004, Taiwan; mingwu@nkust.edu.tw

**Keywords:** workplace spirituality, psychological capital, teacher motivation, elementary school teachers

## Abstract

To improve school quality, it is essential for teachers to play a central role. Teacher efficacy largely depends on strong work motivation, which can be enhanced by fostering workplace spirituality and psychological capital. This study aimed to analyze the demographic differences among elementary school teachers regarding workplace spirituality, psychological capital, and teacher motivation. It also sought to examine the relationships between workplace spirituality, psychological capital, and teacher motivation. Particularly, it aimed to explore the impact of workplace spirituality and psychological capital on teacher motivation. This study included 348 teachers from various elementary schools across Taiwan. Its findings confirmed that the assessments of workplace spirituality, psychological capital, and teacher motivation were both reliable and valid. Male teachers exhibited greater efficacy and resilience in terms of psychological capital compared to their female counterparts. Teachers with more years of experience demonstrated greater efficacy, hope, and psychological capital, while those with fewer years of experience reported higher workplace spirituality. Moreover, workplace spirituality and psychological capital were significantly correlated with teacher motivation. The most influential predictors of teacher motivation were identified as workplace spirituality and psychological capital. As a result, the implication of this study is that school organizations can enhance teacher motivation by promoting workplace spirituality and psychological capital.

## 1. Introduction

Motivation is undeniably crucial for the continuous development of educational systems. According to Ofoegbu (2004) [1], teacher motivation is considered a key factor in promoting school improvement and classroom effectiveness. Also, Somech and Ron (2007) [2] noted that the success of schools is largely determined by teachers’ willingness to exceed expectations and go beyond their prescribed duties. Teacher motivation refers to the desire of teachers to engage actively in the education process and contribute positively to the school environment. It encompasses their attitude toward work, including efforts made to ensure teachers feel happy, satisfied, dedicated, and committed. This motivation enables them to perform at their best, ultimately benefiting students, parents, and society. Additionally, teacher motivation extends to their interest in student discipline and classroom management, which can affect their involvement in both academic and non-academic activities within schools [1]. Thus, examining teacher motivation remains one of the major challenges in the quest for school improvement.

Spirituality is closely connected to a person’s core values, such as love, affection, tolerance, satisfaction, responsibility, and harmonious feelings toward oneself and others (Kaya, 2015) [3], which aligns with the principles of positive psychology. People are increasingly seeking deeper meaning in their jobs and questioning the purpose of their lives and work. Spirituality is an important factor influencing job satisfaction and performance in organizations [4]. As global competition intensifies, employees are no longer viewing work solely as a means to pay the bills; instead, they are seeking a greater sense of life purpose and spiritual fulfillment through their careers [5]. This shift in focus has sparked growing interest in workplace spirituality, giving rise to a new paradigm in organizational science. Elementary school teachers face increased stress and heavier workloads today. By integrating spirituality into the workplace, teachers may experience greater job satisfaction. Satisfied employees tend to have higher motivation and greater commitment to their work and are more likely to remain in their roles [6]. Given that workplace spirituality is known to promote positive work attitudes and behaviors, improve performance, and increase job satisfaction, it is highly probable that workplace spirituality positively influences teacher motivation. Additionally, employees who perceive personal growth, engagement, and productivity and who experience positive emotional states such as pleasure, joy, and energy are better equipped to cope with stress and depression [7]. In the present study, it is expected that employees who perceive a strong alignment with workplace spirituality will also exhibit a high degree of job involvement. Consequently, workplace spirituality is likely to have a positive effect on teacher motivation. Therefore, the purpose of this study was to examine the relationship between workplace spirituality and teacher motivation.

Psychological capital has been demonstrated conceptually and empirically to be a core construct that predicts desirable employee outcomes such as performance and job satisfaction better than other individual resources considered independently [8]. It has also been established that psychological capital leads to better performance and work outcomes and positive work attitudes and behaviors [9]. Psychological capital refers to the state of an individual’s inner life components, which, when combined with personal experiences and resources, collectively determine one’s overall value. These components include efficacy, optimism, hope, and resilience. Viewing positive psychological capacities as resources from which individuals can draw provides a crucial theoretical explanation of how these capacities impact work motivation. Specifically, these psychological resources, fostered by positive emotions, may result in employee attitudes such as emotional engagement [10]. In other words, employees with high psychological capital are more likely to demonstrate strong work motivation. However, there has been limited research on the impact of psychological capital on teacher motivation. Thus, this study aimed to explore the relationship between psychological capital and teacher motivation.

When teachers demonstrate higher levels of workplace spirituality and psychological capital in their approach to teaching, curriculum development, and interactions with students, they are likely to exhibit greater enthusiasm and motivation in performing their school-related tasks. However, empirical research focusing on elementary school teachers and the correlations between workplace spirituality, psychological capital, and teacher motivation remains limited and incomplete. Therefore, the primary aim of this study was to explore the relationships among workplace spirituality, psychological capital, and teacher motivation and to assess the impact of workplace spirituality and psychological capital on teacher motivation.

## 2. Theoretical Framework

### 2.1. The Construct of Teacher Motivation

Teacher motivation encompasses both intrinsic and extrinsic needs. Intrinsic motivation drives teachers to engage in tasks for their own sake and for the satisfaction, sense of achievement, or self-actualization they provide. On the other hand, extrinsic motivation encourages teachers to perform an activity or duty to obtain external rewards, such as a salary. Both intrinsic and extrinsic motivations are crucial for teachers, as they play a significant role in shaping their behavior. As a result, organizations should aim to cultivate and enhance teachers’ intrinsic motivation toward effective teaching while also providing extrinsic motivation to support school improvement initiatives [11]. In this study, teacher motivation was measured in terms of both intrinsic and extrinsic motivation.

### 2.2. The Construct of Workplace Spirituality

Karakas (2010) [12] reviewed 140 articles on workplace spirituality and identified three perspectives on how spirituality supports organizational performance. Workplace spirituality involves employees understanding themselves as spiritual beings whose souls need nourishment in the workplace. It also includes them experiencing a sense of purpose and meaning in their work and finding meaning in task performance [13], as well as experiencing a sense of interconnectedness and community. de Klerk (2005) [14] emphasized that workplace spirituality is reflected in employees’ experiences of meaning, purpose, community, and transcendence within the workplace. Milliman et al. (2003) [15] conceptualized workplace spirituality as encompassing meaningful work, a sense of community, and alignment with organizational values. The Spirituality at Work instrument by Ashmos and Duchon (2000) [16] identified three key dimensions of workplace spirituality: inner life, meaningful work, and community. Based on Ashmos and Duchon (2000) [16] and Milliman et al. (2003) [15], this study measured workplace spirituality using three components: meaningful work, a sense of community, and inner life.

### 2.3. Relationship of Workplace Spirituality with Teacher Motivation

In this study, workplace spirituality refers to experiencing a sense of purpose and meaning in work that goes beyond merely finding meaning in task performance. It emphasizes a deeper connection to one’s work, where the sense of fulfillment comes from a broader, more holistic understanding of purpose. Theoretical assumptions suggest that workplace spirituality may enhance organizational performance, with many postulating a significant positive impact. To date, workplace spirituality has been seen as a means to overcome limitations and create a higher-functioning environment [16,17,18,19]. While empirical research on workplace spirituality is crucial, more attention is needed in this area [14,20]. Workplace spirituality generally has a positive impact on job satisfaction [15,21,22]. Moitreyee and Lalatendu (2022) [23] found a positive relationship between workplace spirituality and teachers’ professional well-being, with positive psychological capital partially mediating this relationship. Milliman et al. (2003) [15] identified five work attitudes influenced by workplace spirituality, emotional organizational commitment, employee retention, internal work contentment, work participation, and institution-based self-worth. Similarly, Pouragha et al. (2022) [24] found that workplace spirituality played a role in improving hospital social workers’ motivation. de Klerk (2005) [14] also highlighted that within a spiritual framework, the construct of meaning in life is crucial for enhancing overall work wellness. It is possible that the quest for meaning in the workplace may lead to increased motivation. Therefore, a link between workplace spirituality and teacher motivation may exist, as teachers who find deeper meaning and purpose in their work are likely to experience greater motivation.

### 2.4. The Construct of Psychological Capital

There is increasing evidence that human resources are vital to organizational success and may provide the highest return on investment for achieving a sustainable competitive advantage [25]. Similarly, in today’s environment, which demands flexibility, innovation, and speed to market, effectively developing and managing employees’ knowledge, experiences, skills, and expertise, collectively referred to as human capital, including psychological capital, have become crucial success factors for sustained organizational performance. The recently recognized major construct of psychological capital, comprising the positive psychological resources of efficacy, hope, optimism, and resilience, has been shown to be associated with various employee attitudinal, behavioral, and performance outcomes [26,27].

Teachers need to possess efficacy to adapt to changes in the school environment and resilience to recover from the setbacks that are likely to occur during the change process. Additionally, to perform effectively, teachers undergoing change need motivation and alternative pathways, defined as hope, when confronted with obstacles. They should also make optimistic attributions when things go wrong and maintain a positive outlook on the future. Gittell et al. (2006) [28] explained that positive relationships can be a source for developing qualities like resilience in the face of change. Further, various studies [8,29,30,31] have identified the positive constructs of efficacy, hope, optimism, and resilience as essential, collectively referring to them as psychological capital. Avey, Wernsing, and Luthans (2008) [27] surveyed 132 employees from a broad cross-section of organizations and jobs, finding that psychological capital, a core factor consisting of hope, efficacy, optimism, and resilience, was related to positive emotions. These emotions, in turn, influenced attitudes (such as engagement and cynicism) and behaviors (such as organizational citizenship and deviance) which are relevant to organizational change. While numerous positive constructs have been studied [32], the four that best align with the definition of psychological capital are efficacy, hope, optimism, and resilience [8,26,27]. Thus, in this study, psychological capital refers to an individual’s positive psychological state of development, characterized by efficacy, hope, optimism, and resilience.

### 2.5. Relationship of Psychological Capital with Teacher Motivation

Researchers have increasingly focused on the impact of positive emotions on physical and psychological well-being in the workplace [33,34,35]. It follows that the psychological resources generated by positive emotions may influence employee attitudes, such as emotional engagement [10], which is characterized by vigor [36]. Luthans et al. (2007) [30] defined psychological capital as a positive psychological capacity, characterized by efficacy, optimism, hope, and resilience. Specifically, Stajkovic and Luthans (2003) [37] incorporated these four capacities, confidence, hope, optimism, and resilience, into their core confidence factor for work motivation. Also, the psychological resources generated by employees experiencing positive emotions may lead to attitudes like emotional engagement (Hodges, 2010) [10], which is characterized by motivation. Özpolat (2022) [38] found a moderately positive relationship between positive psychological capital and both organizational commitment and motivation perceptions. This suggests that psychological capital plays a significant role in enhancing motivation within organizations.

Scheier and Carver (1992) [39] defined optimism as a personal disposition characterized by the expectation that positive outcomes will prevail in the future. Additionally, optimism encompasses cognitive, emotional, and motivational components. As a key component of psychological capital, optimism may be linked to motivation. Herdem (2019) [40] explored the effect of psychological capital on undergraduate students’ motivation and found that resilience, a component of psychological capital, significantly and positively influenced motivation for achievement. This suggests that employees with positive psychological capital are more likely to demonstrate high levels of work motivation. Given this, there is a need for further research into the relationship between psychological capital and teacher motivation. Since little research has been conducted on the impact of psychological capital on teacher motivation, this study specifically aims to explore the relationship between psychological capital and teacher motivation.

## 3. Materials and Methods

### 3.1. Participants

Teachers were recruited from 24 elementary schools located in the southern areas of Taiwan. From each school, approximately 10 to 30 teachers were recruited, resulting in a total sample of 348 teachers, consisting of 93 males and 255 females.

### 3.2. Measures

#### 3.2.1. The Spirituality at Work Scale (SAWS)

The SAWS developed by Ashmos and Duchon (2000) [16] was used to assess the experience of spirituality at work. Workplace spirituality was measured according to the three aspects of meaningful work, a sense of community, and inner life. All the item responses were scored on a 5-point Likert scale, ranging from 1 (strongly disagree) to 5 (strongly agree), with higher scores representing higher workplace spirituality. To test whether this scale could be applied to measuring elementary school teachers, a pilot study was executed with 185 elementary school teachers to test the reliability and validity of the scale. After an exploratory factor analysis of the 18 items, the results showed that of these 18 items, loading on three factors, namely meaningful work, inner life, and a sense of community, each with 6, 4, and 8 items, yielded the largest eigenvalues, accounting for 57.34% of the total variance. The Cronbach alpha of these three subscales was 0.87, 0.84, and 0.85 for the total scale.

#### 3.2.2. The Psychological Capital Questionnaire (PCQ)

The PCQ was developed based on a literature review and the previous studies [8,26,27,41]. According to Luthans et al. (2007) [8], the PCQ, measured by four positive constructs, efficacy, hope, optimism, and resilience, demonstrated high reliability and construct validity. All the item responses were scored on a 5-point Likert scale, ranging from 1 (strongly disagree) to 5 (strongly agree). Higher values indicate more psychological capital. Although this measure of psychological capital has been given previous research attention [8], it is still a relatively new construct. Also, the research subjects and culture are different. To further assess the psychometric properties of the PCQ for this study, a pilot study was conducted with 185 elementary school teachers to evaluate the reliability and validity of the scale. An exploratory factor analysis of the 13 items was performed using the maximum likelihood method with Varimax rotation. The results showed that all the items loaded onto three factors with the largest eigenvalues, accounting for 61.83% of the total variance. The 13-item scale consisted of efficacy, hope, optimism, and resilience. Cronbach’s alpha of these four subscales ranged from 0.82 to 0.88 and was 0.86 for the total scale.

#### 3.2.3. The Teacher Motivation Scale

The Teacher Motivation Scale was developed based on a literature review and prior studies, such as Sanchez-Perkins (2002) [42] and Ofoegbu (2004) [1]. In this present study, teacher motivation includes intrinsic and extrinsic dimensions. All the item responses are scored on a 5-point scale, ranging from 1 (strongly disagree) to 5 (strongly agree). Higher scores indicate greater teacher motivation. Based on Wu (2015) [43], a pilot study was conducted with 410 elementary and secondary school teachers to evaluate the reliability and validity of the scale. After conducting an exploratory factor analysis of the 10 items, it was found that all of the items loaded onto two factors with the largest eigenvalues, accounting for 68.64% of the total variance. Then, the Cronbach’s alpha coefficients were found to be 0.90 and 0.85 for the intrinsic and extrinsic subscales and 0.88 for the total scale. Since the research subjects differed, a pilot study was conducted with 185 elementary school teachers to assess the reliability and validity of the scale. An exploratory factor analysis of the 10 items was performed using the maximum likelihood method with Varimax rotation. The analysis revealed that all the items loaded onto two factors with the largest eigenvalues, accounting for 59.23% of the total variance. The 10-item scale consisted of intrinsic and extrinsic motivation. The Cronbach’s alpha coefficients for these two subscales were 0.84 and 0.80, while the total scale had a Cronbach’s alpha of 0.84.

### 3.3. Design and Analyses

First of all, this study further examined the psychometric properties of the three scales for this study. So, the reliability and validity of the three scales were tested. Cronbach’s α coefficients were utilized to test the reliability of the scales, and principal component analyses were used to test the validity of the scales.

Second, this study aimed to compare the demographic variable differences in elementary school teachers in terms of workplace spirituality, psychological capital, and teacher motivation. A Multivariate Analysis of Variance (MANOVA) design was formulated to test the demographic differences in workplace spirituality, psychological capital, and teacher motivation. Next, this study aimed to explore the relationships among workplace spirituality, psychological capital, and teacher motivation. Intercorrelation matrices among all the variables were computed. Finally, a stepwise multiple regression analysis was conducted to determine whether both workplace spirituality and psychological capital were the best predictors of teacher motivation.

## 4. Results

### 4.1. Differences in the Demographic Variables of Elementary School Teachers Regarding Workplace Spirituality, Psychological Capital, and Teacher Motivation

A Multivariate Analysis of Variance compared the mean scores of the demographic variable (gender, age, education, and years of working experience) differences in teachers in terms of their scores for workplace spirituality, psychological capital, and teacher motivation. Only the tests for gender (Wilks’ λ = 0.92, *F* = 2.64) and years of working experience (Wilks’ λ = 0.82, *F* = 1.58) were significant (*p* < 0.05). Subsequently, univariate F tests were conducted to identify which specific variables contributed to the overall multivariate significance. Since there were only significant variable differences in the elementary teachers in terms of workplace spirituality and psychological capital based on their gender and years of working experience, the means, standard deviations, and univariate *F* tests for variable differences in the teachers in terms of workplace spirituality and psychological capital based on gender and years of working experience are presented in Table 1.

Gender-based differences were significant only in terms of efficacy and resilience of psychological capital. Based on the mean values, male elementary teachers scored higher on efficacy and resilience than female teachers. Years of working experience determined significant differences in the three dimensions of psychological capital, efficacy, hope, optimism, and the total scale for psychological capital. Also, years of working experience determined significant differences in all three aspects of workplace spirituality, meaningful work, inner life, sense of community, and the total scale for workplace spirituality. According to Scheffe’s post-test, elementary school teachers with over 21 years of experience demonstrated greater efficacy, hope, and psychological capital, while those with less than 5 years of experience reported higher levels of meaningful work, inner life, sense of community, and workplace spirituality.

### 4.2. Correlations of Teacher Motivation with Workplace Spirituality and Psychological Capital

As shown in Table 2, both workplace spirituality and psychological capital were strongly related to teacher motivation. Higher workplace spirituality and greater psychological capital corresponded with higher teacher motivation. We can conclude that workplace spirituality and psychological capital were the most robust indicators of teacher motivation.

Stepwise multiple regression analyses were conducted to determine whether both workplace spirituality and psychological capital could predict teacher motivation. Two types of criterion variables were entered separately. The first type of predictors included three dimensions of workplace spirituality and four dimensions of psychological capital, while the criterion variables encompassed two dimensions of teacher motivation. The second type of predictors consisted of the total scale for workplace spirituality and the total scale for psychological capital, with the criterion variable being the total scale for teacher motivation. The results of the analyses for predicting teacher motivation are summarized in Table 3.

Our findings revealed that meaningful work and a sense of community within workplace spirituality, along with optimism and hope within psychological capital, were significant predictors of intrinsic motivation. Additionally, a sense of community within workplace spirituality and hope within psychological capital emerged as the significant predictors of extrinsic motivation. Moreover, both the total scales for workplace spirituality and for psychological capital jointly predicted the total scale for teacher motivation.

## 5. Discussion

The measures of workplace spirituality, psychological capital, and teacher motivation were confirmed to be both reliable and valid, establishing their effectiveness as essential tools for assessing school organizations and teachers. Additionally, male elementary school teachers showed higher levels of perceived efficacy and resilience in psychological capital compared to their female counterparts. Teachers with more years of experience exhibited higher levels of perceived efficacy, hope, and psychological capital, whereas those with fewer years of experience displayed greater workplace spirituality. Statistically significant correlations were found among workplace spirituality, psychological capital, and teacher motivation. Ultimately, workplace spirituality and psychological capital emerged as the most robust indicators of teacher motivation.

It is a well-established finding that teachers often report lower motivation and higher stress levels compared to other professional groups [44,45]. Pihie and Elia (2004) [46] have indicated that teachers cite factors such as workload, pressure, and unsatisfactory leadership as reasons for not favoring the teaching profession. In the context of teaching and teacher education, motivation plays a vital role in influencing several factors, including what draws individuals to the teaching profession, how long they persist with their initial teacher education courses, their commitment to the teaching profession, and their level of engagement in both their work and profession [47]. Specifically, motivation stands out as the most important psychological factor influencing work performance efficiency [48]. Contented workers with high motivation and job commitment are more likely to remain in their job roles [6]. As a result, teacher motivation is regarded as a key factor in enhancing classroom effectiveness and driving school improvement [1].

Given interest is growing in incorporating spirituality into the workplace (Delbecq, 2005) [49], the spiritual quest associated with positive psychology became a key focus of this study. As anticipated, workplace spirituality emerged as one of the most important predictors of teacher motivation. This finding supported the study conducted by de Klerk (2005) [14], which highlighted the significance of a spiritual framework and the construct of meaning in life in enhancing work wellness through an individual’s sense of life purpose. It also corroborated previous research indicating that workplace spirituality generally exerts a positive impact on job satisfaction and overall work performance [4,15,21,22]. Particularly, it was consistent with the findings of Pouragha et al. (2022) [24], which indicated that workplace spirituality could enhance motivation. Thus, by incorporating spirituality into the workplace, teachers experience increased teacher motivation and job satisfaction.

Another significant indicator of teacher motivation was psychological capital, a concept consistent with several studies demonstrating that the core components of psychological capital, including efficacy, hope, optimism, and resilience, were associated with various employee attitudes, behaviors, and performance outcomes [26,27]. Sridevi and Srinivasan (2012) [9] pointed out that psychological capital contributes to improved performance, favorable work outcomes, and positive work attitudes and behaviors. Notably, research by Hodges (2010) [10] and Özpolat (2022) [38] indicated a link between psychological capital and motivation. Therefore, a higher level of positive psychological capital is likely to result in increased teacher motivation.

In sum, since teachers play a vital role in the quality of teaching, student motivation, school success, and the implementation of educational reform, it is crucial for educational leaders to recognize the importance of teacher motivation. Motivated teachers are essential for keeping students motivated and promoting high-quality education. Schuitema et al. (2016) [50] stressed that motivated teachers not only demonstrate a high quality of teaching but also inspire student learning. The findings of this study revealed that both workplace spirituality and psychological capital were strongly linked to teacher motivation. It was concluded that elementary school teachers with greater workplace spirituality and psychological capital exhibited greater motivation. In other words, fostering workplace spirituality and psychological capital in an educational context enhances teachers’ willingness to work with joy and dedication to the benefit of others rather than solely for personal gain. These results suggest that school and authorities should focus on promoting factors that enhance workplace spirituality and psychological capital, as these factors lead to increased teacher motivation, improved school performance, and better student learning outcomes. The implication of this study for school organizations is that they should actively encourage the development of workplace spirituality and psychological capital among teachers, as this can evoke teacher motivation and contribute to a school’s competitive advantage. While this study involved 348 teachers recruited from southern Taiwan, future research could expand the sample size to increase its external validity and also explore additional factors that influence teacher motivation.

## Figures and Tables

**Table 1:** Means, standard deviation, and univariate *F* tests of gender and years of experience of teachers in terms of psychological capital and workplace spirituality.

Variable	Demographic Variable	M	SD	*F*	Scheff’e
	Gender				
Efficacy	(1) Male	4.01	0.45	7.70 **	
	(2) Female	3.86	0.43		
Resilience	(1) Male	3.99	0.44	4.38 *	
	(2) Female	3.85	0.44		
	Years of working experience				
Efficacy	(1) Below 5 years	3.97	0.56	3.42 **	(5) > (2)
	(2) 6–10 years	3.74	0.44		
	(3) 11–15 years	3.85	0.42		
	(4) 16–20 years	3.89	0.39		
	(5) Over 21 years	4.01	0.44		
Hope	(1) Below 5 years	4.04	0.44	3.93 **	(5) > (3)
	(2) 6–10 years	3.79	0.44		
	(3) 11–15 years	3.82	0.49		
	(4) 16–20 years	3.90	0.46		
	(5) Over 21 years	4.04	0.47		
Optimism	(1) Below 5 years	3.93	0.55	2.94 *	ns
	(2) 6–10 years	3.66	0.49		
	(3) 11–15 years	3.64	0.56		
	(4) 16–20 years	3.79	0.53		
	(5) Over 21 years	3.85	0.54		
Total Scale of Psychological Capital	(1) Below 5 years	3.96	0.41	3.91 **	(5) > (3)
	(2) 6–10 years	3.75	0.36		
	(3) 11–15 years	3.79	0.40		
	(4) 16–20 years	3.87	0.39		
	(5) Over 21 years	3.97	0.42		
Meaningful Work	(1) Below 5 years	4.12	0.45	4.06 **	(1) > (2)
	(2) 6–10 years	3.72	0.41		(1) > (3)
	(3) 11–15 years	3.76	0.49		(1) > (4)
	(4) 16–20 years	3.74	0.62		
	(5) Over 21 years	3.84	0.57		
Inner Life	(1) Below 5 years	4.29	0.58	2.73 *	ns
	(2) 6–10 years	4.02	0.49		
	(3) 11–15 years	4.02	0.47		
	(4) 16–20 years	4.01	0.52		
	(5) Over 21 years	4.11	0.47		
A Sense of Community	(1) Below 5 years	4.00	0.44	2.57 *	ns
	(2) 6–10 years	3.70	0.38		
	(3) 11–15 years	3.71	0.44		
	(4) 16–20 years	3.77	0.51		
	(5) Over 21 years	3.78	0.55		
Total Scale of Workplace Spirituality	(1) Below 5 years	4.10	0.38	4.17 **	(1) > (2)
	(2) 6–10 years	3.78	0.33		(1) > (3)
	(3) 11–15 years	3.80	0.39		(1) > (4)
	(4) 16–20 years	3.81	0.49		
	(5) Over 21 years	3.87	0.45		

Note. N = 348. ns = non-significant. * *p* < 0.05 ** *p* < 0.01.

**Table 2:** Correlations of teacher motivation with workplace spirituality and psychological capital.

Variable	Intrinsic Motivation	Extrinsic Motivation	Total Scale of Teacher Motivation
Meaningful Work	0.75 **	0.31 **	0.63 **
Inner Life	0.49 **	0.23 **	0.43 **
Sense of Community	0.58 **	0.43 **	0.60 **
Total Scale of Workplace Spirituality	0.73 **	0.40 **	0.67 **
Efficacy	0.45 **	0.33 **	0.47 **
Hope	0.56 **	0.39 **	0.56 **
Optimism	0.60 **	0.27 **	0.52 **
Resilience	0.50 **	0.25 **	0.44 **
Total Scale of Psychological Capital	0.65 **	0.38 **	0.61 **

Note. N = 348. ** *p* < 0.01.

**Table 3:** Summary of stepwise multiple regression for workplace spirituality and psychological capital predicting teacher motivation.

Criterion Variable	Predictor Variable	Beta	Multiple R	Multiple R^2^	t
Intrinsic Motivation	Meaningful Work	0.50	0.75	0.56	10.54 **
	Optimism	0.17	0.78	0.60	3.82 **
	Hope	0.15	0.79	0.62	3.70 **
	Sense of Community	0.13	0.80	0.63	2.98 **
Extrinsic Motivation	Sense of Community	0.33	0.43	0.19	6.25 **
	Hope	0.25	0.49	0.24	4.74 **
Total Scale of Teacher Motivation	Total Scale of Workplace Spirituality	0.48	0.67	0.45	9.30 **
	Total Scale of Psychological Capital	0.30	0.71	0.50	5.88 **

Note. Multiple R and multiple R^2^ (cumulative values). ** *p* < 0.01.

## Data Availability

The data can be made available upon request.
